# Smiles2Monomers: a link between chemical and biological structures for polymers

**DOI:** 10.1186/s13321-015-0111-5

**Published:** 2015-12-29

**Authors:** Yoann Dufresne, Laurent Noé, Valérie Leclère, Maude Pupin

**Affiliations:** Univ. Lille, CNRS, Centrale Lille, UMR 9189-CRIStAL-Centre de Recherche en Informatique Signal et Automatique de Lille, 59000 Lille, France; Inria Lille Nord Europe, Bonsai team, Parc scientifique de la Haute Borne, 40 avenue Halley, 59650 Villeneuve d’Ascq, France; Univ. Lille, INRA, ISA, Univ. Artois, Univ. Littoral Côte d’Opale, EA 7394 - ICV - Institut Charles Viollette, 59000 Lille, France

**Keywords:** Polymer, Monomer, Peptide, Chemical structure, Compound search, Algorithm, Graph

## Abstract

**Background:**

The monomeric composition of polymers is powerful for structure comparison and synthetic biology, among others. Many databases give access to the atomic structure of compounds but the monomeric structure of polymers is often lacking. We have designed a smart algorithm, implemented in the tool Smiles2Monomers (s2m), to infer efficiently and accurately the monomeric structure of a polymer from its chemical structure.

**Results:**

Our strategy is divided into two steps: first, monomers are mapped on the atomic structure by an efficient subgraph-isomorphism algorithm ; second, the best tiling is computed so that non-overlapping monomers cover all the structure of the target polymer. The mapping is based on a Markovian index built by a dynamic programming algorithm. The index enables s2m to search quickly all the given monomers on a target polymer. After, a greedy algorithm combines the mapped monomers into a consistent monomeric structure. Finally, a local branch and cut algorithm refines the structure. We tested this method on two manually annotated databases of polymers and reconstructed the structures de novo with a sensitivity over 90 %. The average computation time per polymer is 2 s.

**Conclusion:**

s2m automatically creates de novo monomeric annotations for polymers, efficiently in terms of time computation and sensitivity. s2m allowed us to detect annotation errors in the tested databases and to easily find the accurate structures. So, s2m could be integrated into the curation process of databases of small compounds to verify the current entries and accelerate the annotation of new polymers. The full method can be downloaded or accessed via a website for peptide-like polymers at http://bioinfo.lifl.fr/norine/smiles2monomers.jsp.

**Graphical abstract:**
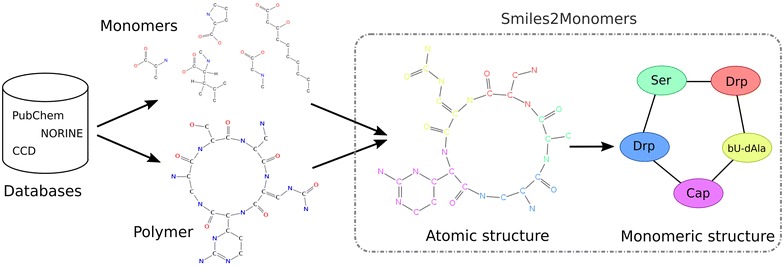
.

**Electronic supplementary material:**

The online version of this article (doi:10.1186/s13321-015-0111-5) contains supplementary material, which is available to authorized users.

## Background

Chemists (pharmacists) and biologists study natural products because they are a valuable source of biologically active molecules. The most promising secondary metabolites are synthesized by huge enzymes assembling building blocks, called monomers, into small polymers. Chemical structures of the molecules are obtained by techniques such as mass spectrometry. Activity or toxicity of the molecules are tested experimentally against various targets. Synthesis pathways are investigated to better understand the complete process and be able to design desire products. More recently, bioinformatics genome mining outputs possible secondary metabolites produced by a targeted species. The best predicted metabolites are nonribosomal peptides because, most often, a co-linearity is observed between the domains predicted in the producing enzymes and the monomers incorporated in these polymers. The approaches mentioned above are complementary and enrich the knowledge on these secondary metabolites. But, to our knowledge, no freely available cheminformatics or bioinformatics tool provides an automatic connection from chemical (atomic structure) to biological (monomeric structure) representations of the compounds. In this article, we present an efficient and accurate algorithm that infers the monomeric structure of a polymer from its atomic structure. It is implemented in the Smiles2Monomers abbreviated s2m tool (http://bioinfo.lifl.fr/norine/smiles2monomers.jsp).

The s2m tool was developed to help the determination of the monomeric structure of nonribosomal peptides (NRPs) from their chemical structure given in scientific articles. This determination is mandatory for the input of a new NRP in the Norine database [1]. However, the applications are numerous. First, other databases can rely on s2m to increase the accuracy of their data. For example, the Protein Data Bank (PDB) carried out a remediation process concerning the small molecules [2] and peptide-like compounds [3] stored in PDB entries. This process ends up with the remediation of the CCD and the creation of the Biologically Interesting molecule Reference Dictionary (BIRD) which contains the polymeric structures of peptides. A different application is the analysis of compounds by tools dedicated to monomeric structures to, for example, compare polymers [4] or predict polymer activities [5]. These tools complement cheminformatics tools that analyze the atomic structures of polymers. With the same idea, s2m can identify fragments designed by other tools or atomic structures extracted from a database. Indeed, several cheminformatics tools rely on chemical substructures, also called fragments. The presence or absence of a list of fragments in a given molecule can be summarized in an array called fingerprint, reducing the algorithmic complexity needed to compare two molecules or search for a molecule in a database (for a recent review see [6]). These structural features extended by other molecular descriptors such as 3D or physico-chemical properties are also applied in Quantitative Structure-Activity Relationship (QSAR) methods to predict compounds activity (for recent reviews, see [7, 8]). The determination of relevant molecular descriptors is the core of QSAR modeling and is still an ongoing work. s2m can contribute to this challenge.

Only a few methods have been described to recognize simple monomeric structures from atomic structures. The O’Donnell et al.  [9] method was designed to deduce the sequence of a given polymer type, based on the definition of all the unique atoms of the monomers and the description of the functional groups implied in the polymer bonds. This method was illustrated on proteinogenic peptides. In another method, called CHUCKLES [10], monomers are matched sequentially against the atomic structure of the polymer by decreasing size (number of atoms). Therefore, a monomer can match by chance and take the position of the smaller but accurate one. So, these methods will certainly fail to infer the correct structure of intricate polymers. For example, nonribosomal peptides contain amino acid derivatives or other types of monomers. The chemical bonds between the monomers can be peptide bonds, glycosidic bonds or oxidative cross-linking leading to non-linear monomeric structures. Some monomers are able to form up to five bonds. In these cases the algorithm needs to be smarter by exploring several solutions. Moreover, the O’Donnell et al. and CHUCKLES methods are not distributed in a free program. Otherwise, other tools [11–13] break polymers into fragments that are not identified monomers. For example, these tools infer a set of fragments by extracting frequent common patterns between molecules in given databases. This strategy, known as deconstruction, generates fragments that can then be linked to reconstruct new drug-like molecules in the fragment-based drug design approach (see [14] for review). Finally, our algorithm identifies the monomers occurring in a target polymer based on a database of monomers. This strategy enables to obtain the name of the determined fragments. We design dedicated algorithms and data representation for the two steps of s2m: first the monomers of the database are mapped on the atomic structure, second the best tiling is computed so that non-overlapping monomers cover all the polymer structure.

The first step, which is the monomer search, corresponds to the following two common graph theory problems: “Maximum Common Subgraph” (MCS) problem [15] and “Subgraph Isomorphism” (SI) problem [16]. Indeed, atomic structures can be represented as graphs in which nodes are labeled by atom names and edges are the covalent bonds. So, searching for a monomer in a polymer is searching for a small graph in a larger one. In the MCS problem, the goal is to find the largest common part between two graphs (here a monomer and a polymer). Many algorithms in cheminformatics or graph theory exist to solve the MCS [17–21] (see [22] for a review on MCS for bioinformatics), but MCS is proved NP-Complete [15]. Exact algorithms to solve it are slow in practice when the number of labels (atom names) is small. In the SI problem, the goal is to find where a given graph (a monomer) can map on another larger graph (a polymer). Many algorithms exist to solve the SI problem in general [23] or for specific classes of graphs [24]. Cheminformatics libraries such as CDK [25] and OpenBabel [26] use the VF2 algorithm [27]. This algorithm is still exponential due to the NP-completeness of the problem but is faster than the Ullman one in practice. We designed an efficient strategy to optimize the search, with a fast branch and bound algorithm. We optimized the VF2 algorithm with the strategies presented in Shang publication [28] and used by Zhu for the search of molecular graphs into molecular databases [29]. The novelty of our SI algorithm is the computation of indexes by dynamic programming to construct the best Markovian chain that minimizes the search time. On its first step, our algorithm guarantees finding all the occurrences of any given monomer in a polymer.

The second step, which is the monomer tiling, is the main contribution of our method. The objective is to maximize the number of atoms of the polymer covered by monomers (tiles), without overlap between the tiled monomers. A naïve strategy could be to try all the possible subsets of tiles and to choose the best one. But, this strategy is factorial in time, i.e. more than exponential, as a function of the number of tiles (that could be hundreds), which is not acceptable in practice. Another possibility could be to create a compatibility graph between the tiles (tiles are compatible if they do not overlap) and to search for all cliques maximizing the coverage of the polymer. Unfortunately, finding all maximal cliques is an NP-Complete problem [30] and our compatibility graphs would be too large to enumerate all possibilities. In the O’Donnell et al. [9] and CHUCKLES [10] articles, the tiling and the isomorphism are executed simultaneously. The monomers are tiled while they are mapped. So, an atom already assigned to the monomer *m*1 cannot be assigned to another monomer *m*2, even if the first assignment is false. In practice, this method works for small monomer databases by performing the isomorphism monomer by monomer in decreasing atomic size order. But, when the number of monomers exceed hundreds and when the database contains similar monomers such as amino acid derivatives, this greedy method will fail. So, we created a method to refine a greedy pre-solution by successive and local branch and cut algorithms. In the second step, our algorithm outputs an adequate solution, but not necessarily the exact one.

The main steps of s2m method will be described with a focus on the tiling approach. Then, the results obtained on two manually curated databases are presented and discussed.

## Materials

s2m uses monomer databases to find monomeric structure of target polymers. To determine the performance of s2m, the tested datasets need to match the following constraints: polymers must be described by their chemical and monomeric structures. Monomers must also be described by their chemical structure. So, we extracted two sets of small biological compounds from manually curated databases.

### Norine: a dataset of nonribosomal peptides to test s2m

Norine [1] is a database containing nonribosomal peptides (NRPs), represented by their monomeric structure. These peptides display challenging features that complicate the monomeric decomposition: they are composed not only of amino acids (including derivatives and non-proteinogenic ones), but also carbohydrates and lipids ; their monomers are linked not only by peptide bonds but also by oxidative cross-linking and by glycosidic bonds, among others. We automatically extracted chemical structures from PubChem [31] for a set of 327 NRPs.

### Chemical Component Dictionary: a dataset of small molecules to test s2m

The Chemical Component Dictionary (CCD) [32] contains all the residues and the small molecule components found in some Protein Data Bank (PDB) entries. PDB [33] is the major worldwide resource of 3D structures of biological macro-molecules. The CCD dictionary provides, among other annotations, SMILES representation for each small component present in PDB. Among the components stored in CCD, some polymers have detectable internal substructures, and the list of their sub-components is included in their entry. We extracted from CCD 378 components with documented internal substructures.

The two datasets are independent. Indeed no polymer of Norine is also present in CCD. In their respective monomer databases, only 61 are in common (for more than 500 in each monomer dataset) and in most of the cases they are classical compounds like the 20 proteinogenic amino acids. This non-overlapping between the two datasets allows us to test the algorithm into two different contexts, so to prove that s2m is efficient on different polymer types.

For any dataset, s2m indexes a monomer database to increase speed and accuracy. Three inputs are required for this preliminary step. The data are stored in text files written in JSON format in which the atomic structures are represented by the commonly used line notation for chemical structures called SMILES [34].

### First prerequisite: monomers to annotate the target polymer

The monomeric structure is inferred by mapping given monomers to the atomic structure of a target polymer. The monomer database can be either dedicated to the type of target polymers, if known, or general with all types of monomers. For example, if a peptide is submitted, only the 20 proteinogenic amino acids are needed in the database. s2m tool supplies precomputed databases, but users can also submit their own. The 529 monomers stored in Norine were used on the Norine dataset, while the 506 sub-components parts of the CCD components with documented internal substructures were used on the CCD dataset.

### Second prerequisite: chemical reaction rules to generate residues

As monomers are truncated during chemical reactions while incorporated in the polymer, s2m needs to delete the lost atoms before searching for them in the polymer. The truncated monomers are called *residues*. So, another input of the software is a list of chemical reaction rules that are atoms lost by functional groups potentially altered while bonded with other monomers. The corresponding reactions also depend on the type of target polymer. For example, amino acids are bonded by peptide bonds between amino and acid moiety or by disulfide bonds between two thiol groups. We built the chemical reaction rules by studying the synthesis of the polymers stored in the operated databases. So, s2m tool supplies a set of rules describing the most frequent bonds observed in biological polymers.

### Third prerequisite: polymers to construct selective indexes

To construct selective indexes, frequencies of parts of the monomers are calculated on a representative polymer database. For example, indexes for the amino acids should be learned on a database of peptides. For each tested dataset, the learning database was a subset of its own polymers.

## Methods

**Fig. 1:**
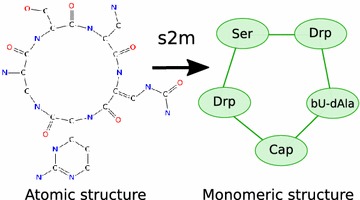
s2m goal: connecting the biological representation to the chemical representation The monomeric structure of a polymer is inferred from its atomic structure

Given the atomic structure of a polymer and all the candidate monomers for a given type of polymer, our goal is to extract its monomeric structure (see Fig. 1). To reach this goal, we split the monomeric structure inference problem into two disjoin steps that are solved sequentially. First, each candidate residue is independently searched on the polymer and then, the mapped residues are tiled against the polymer to find an optimal coverage of its atoms. As the monomer database is an input of s2m, a smart index is built during a preliminary step, to optimize the first step.

### Step 0: indexation to minimize the search time

To begin, all possible residues are generated from the monomers of the given database. Indeed, the monomers are not totally included in the polymer, but they lose atoms during the chemical reactions leading to the polymer. The truncated monomers are called *residues*. All residues generated are searched for in the target polymer. As the number of residues can exceed hundreds and as they are generated once before the searches, we designed an efficient strategy to optimize the search. The main idea is to start with parts of the searched molecules that are rare in the target molecules. So, an index is built by ordering the atoms of the searched molecules. A molecule index starts by a chosen atom (label) and extends it with neighbors atoms recursively to construct successive sub-patterns. To be even more selective, when toolkits like CDK [25] look for the first most selective label [35], we propose to extend the reasoning to all the labels. We design Markovian chains that construct the most selective succession of sub-patterns. This succession minimizes the time of the isomorphism of step 1. More details about the method to create and search the chains are provided in Additional file 1.

### Step 1: search of all monomers to cover the polymer

As explained before, residues are generated from monomer databases by applying the predefined chemical reaction rules. Most of the monomers can form at least two bonds with other monomers, but all these bonds are not always formed when a monomer is integrated into a polymer. So, all the possible residues must be generated from a given monomer by applying recursively the chemical reaction rules. The residues generated from a given monomer are ordered in a Directed Acyclic Graph (DAG) that constitutes a family.

**Fig. 2:**
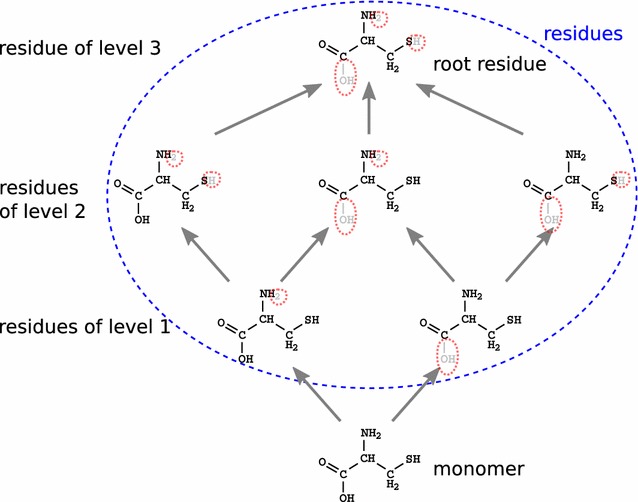
Directed Acyclic Graph representing the cysteine family Creation of the cysteine family using three rules (peptide bond from NH2, peptide bond from C(=O)OH and SH bond)

For example in Fig. 2, the full cysteine monomer first loses atoms during the creation of a peptide bond from the amino or acid moiety. Two residues are generated at this first level. The chemical reaction rule for the disulfide bond is not applied at the first level because this bond cannot occur alone. Then, another chemical reaction is applied on each previous residue: the peptide bond for the other moiety or the disulfide bond. Three more residues are generated at this second level. To finish, the third and last chemical reaction rule is applied to each residue. A single residue is generated at this third and last level. This residue is called the root residue as it is the smallest one (with the lowest number of atoms). So, six residues are generated from the initial cysteine monomer by applying three chemical reaction rules.

Because the residues are generated by applying successive chemical reaction rules, a residue of level *i* is totally included in its parent residues of level $i-1$. So, if a residue of level *i* is not found in a target polymer, then none of its parents can be found in this polymer. Based on these properties, an index (see Additional file 1) corresponding to the root residue is constructed. This index can then be searched efficiently on a target polymer with a branch and bound subgraph isomorphism algorithm. If the root residue is found, its descendant residues are recursively searched for (extensions of few atoms from the children matches), following the DAG of the residues family.

The performance of our algorithm heavily depends on the number of node labels. Indeed, when the number of node labels increases, the computation time decreases. For the natural organic molecules, around nine atoms are observed (C, H, O, N, S, P, I, Cl and Br). So, atomic graphs contain few different node labels. To increase the number of node labels, we transform the chemical structures by graphs of bonds, also called *line graphs* [36] because edges became nodes. A node label is thus composed of two non-hydrogen atoms and their bond (see next paragraphs for a more precise description). Inside the nodes, we keep the origin of each atom to construct explicit and unambiguous structures that distinguish similar structures of different molecules (like cyclopropane and isobutane). This raises the number of labels from *n* to more than $n^2$, with *n* representing here the number of non-hydrogen atoms of the molecule. Increasing the number of labels improves the selectivity of each label but raises the average arity of each node. Globally, the computational time is reduced and more relevant indexes are constructed.

Note that our matching method is flexible: the node labels (chemical bonds in our case) can be compared with different matching functions. We implemented an exact function called *strict matching* and a function tolerating some errors called *light matching*.

**Fig. 3:**
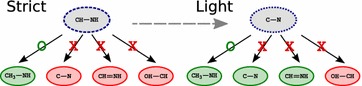
Strict and light matching rules. A bond in a monomer represented in a strict or light matching search strategy compared to four examples of bonds in a polymer with their respective matching results

With the strict matching, atoms, bond multiplicity and numbers of hydrogen partners are compared all at once between the query and the target bonds. The part (a) of Fig. 3 shows possible matches and mismatches of a query bond of a residue on a polymer bond. The bond in the polymer must be single, while *C* and *N* must be linked to at least one hydrogen each. So, only one bond of the polymer matches the residue bond.

With the light matching, bond multiplicity or aromaticity and numbers of hydrogen partners can differ. This matching enables moves of protons and electrons, enabling the recognition of, for example, tautomeric molecules. The light matching removes all hydrogen atoms and bond multiplicity from the query bond. Part (b) of Fig. 3 illustrates the increase in the number of matches due to the light matching. Bonds with no hydrogen or double bonds in the polymer match with the query bond.

The strict matching function is fast because of the selectivity of each node label. It maps the majority of the monomers in the studied polymers. The light matching function is about 100 times slower than the strict because it is less selective. Light matching is therefore useful for difficult compounds with topology changes such as tautomery. It can also match residues forming unusual bonds.

### Step 2: monomer tiling to construct the monomeric structure

The monomer search outputs many overlapping residues mapped on the target polymer. The monomer tiling is the choice of the best candidates, among these puzzle tiles, that create the most relevant puzzle of the polymer. We call this selection the tiling step. Our goal is to select a subset of mapped residues (more generally tiles) that maximizes the number of atoms of the polymer covered by residues (called the coverage) and avoids overlaps between residues (no atom in common between two residues).

#### Greedy tiling algorithm to quickly approximate a solution

Because best solutions cannot be computed in reasonable time, we chose to quickly approximate a solution using a greedy tiling algorithm and to refine this pre-solution. Our greedy tiling algorithm sorts mapped tiles using Occam’s razor principle. The following criteria are used :

1. The larger tiles (larger number of atoms), the better because they have less chances to map randomly on the target polymer than the smaller ones.
2. For tiles of the same size, the smaller number of attachment points, the better because this is more probable in real polymers.
3. For same number of attachment points, the more frequent bonds linking the residue to others, the better because polymers are mainly synthesized by one (or few) major type(s) of bonds and less frequent other types. For example, nonribosomal peptides are synthesized by enzymes creating peptide bonds between amino acids and, occasionally, secondary enzymes add other monomers linked by other types of bonds. The priority of the bonds is defined within the chemical reaction rules prerequisite.

If the prerequisites are adapted to the target polymer, the greedy tiling algorithm provides a full coverage most of the time.

#### Modulation and complementary tilings to optimize the coverage

When the polymer is partially covered, the light matching is applied to the regions that remain uncovered and another tiling is performed. The strategy to fill in these regions is based on the possible causes of the partial covering, which could be the following:

- Selection of too large monomers that cover regions they should not. So, their correct neighbors cannot map where they should.
- Mismatch of the correct monomer because of tautomery or other little differences in the structure representation.
- Lacking of the correct monomer in the database.

Moreover, uncovered regions are often partially occupied by small monomers mapping by chance. To solve these problems, we start with the tiling obtained by the monomer search and try to refine it. First, the tiles neighboring uncovered regions are removed. Then, we run a light monomeric search on uncovered regions. Performing the search on these neighborhood regions reduces the computation time. As in the first step, a greedy tiling is performed after the new mapping. In most cases, this second tiling solves the coverage problem. For the small number of cases where uncovered regions remain, we use a local branch and bound algorithm to refine the solution and try to find the correct tiles.

**Fig. 4:**
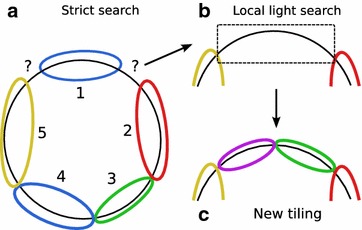
Modulation. The polymer represented by the black circle is partially covered by 5 residues (**a**). To find the complete coverage we perform a local search step with the light search function  (**b**) before another tiling step  (**c**) and a branch and cut step to refine if it is necessary

For example in part (a) of Fig. 4, the polymer have uncovered regions after the execution of the greedy tiling algorithm. By observing the situation, one can suppose that residue 1 was mapped by chance in the uncovered region. So, we can suppose that this residue is not really present in the polymer. The more likely scenario is that the correct monomers were not mapped during the strict search. Then, a smaller residue randomly mapped on this uncovered region and was tiled in it. In part (b) of Fig. 4, the neighbors of the uncovered region are removed to run the light search on a larger uncovered region. Finally, new and correct residues are tiled, as illustrated in part (b) of the figure. The new tiling outputs a totally covered polymer.

## Results and discussion

As mentioned in Materials part, we use two independent sets, the Norine database and the Chemical Component Dictionary (CCD) to validate the s2m tool. We run s2m on polymers of each set, using their respective monomer databases and chemical reaction rules designed by ourselves. The aim was to compare the monomeric structures obtained by s2m with the manually annotated ones. The results can be consulted on: http://bioinfo.lifl.fr/norine/smiles2monomers.jsp.

### s2m reaches an average correctness rate of 0.943 per polymer

For each polymer, we calculated the *coverage rate* defined by the number of atoms in covered regions over the total number of atoms. In theory, this rate should be 1 for all polymers as we had all the monomers corresponding to the studied polymers and as the monomeric structure was determined manually. As we knew the real polymeric structure, we were able to calculate *the correctness* of s2m predictions. *The correctness rate* is the number of atoms in the monomers that are identical in the predicted and curated monomeric structures over the total number of atoms. The two rates allow us to distinguish the 3 following classes:

- True positive (TP): coverage of 1 and correctness of 1
- False positive (FP): coverage of 1 and less than 1 in correctness
- False negative (FN): less than 1 in coverage and correctness.

**Fig. 5:**
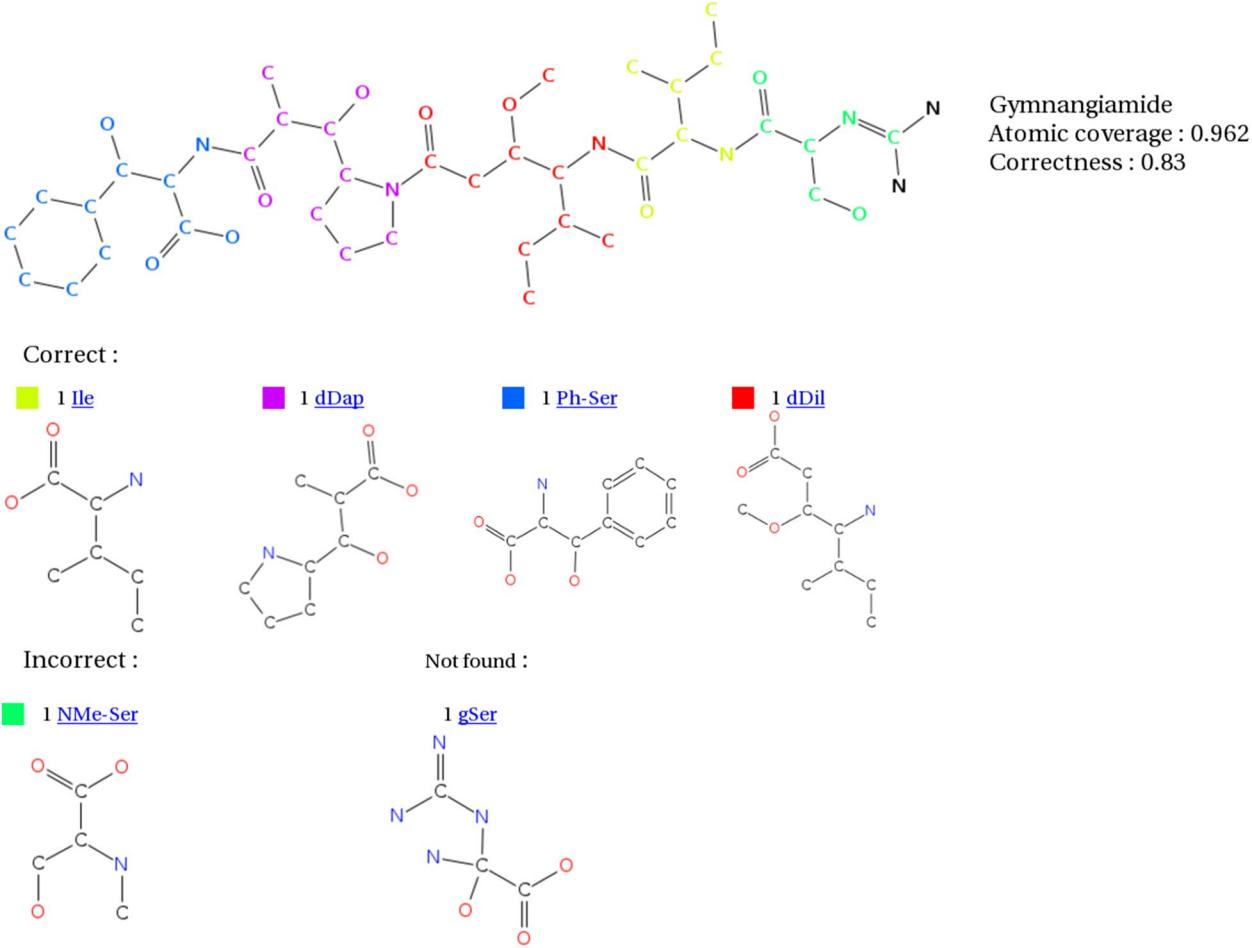
Gymnangiamide analyzed by s2m before SMILES corrections. The execution of s2m on the Norine database allows us to discover wrong annotations in the data. On this example the gSer SMILES was wrong so the software was not able to detect it. The NMe-Ser monomer find in the polymer is an artifact due to a smaller random match on the uncovered region let by gSer

**Fig. 6:**
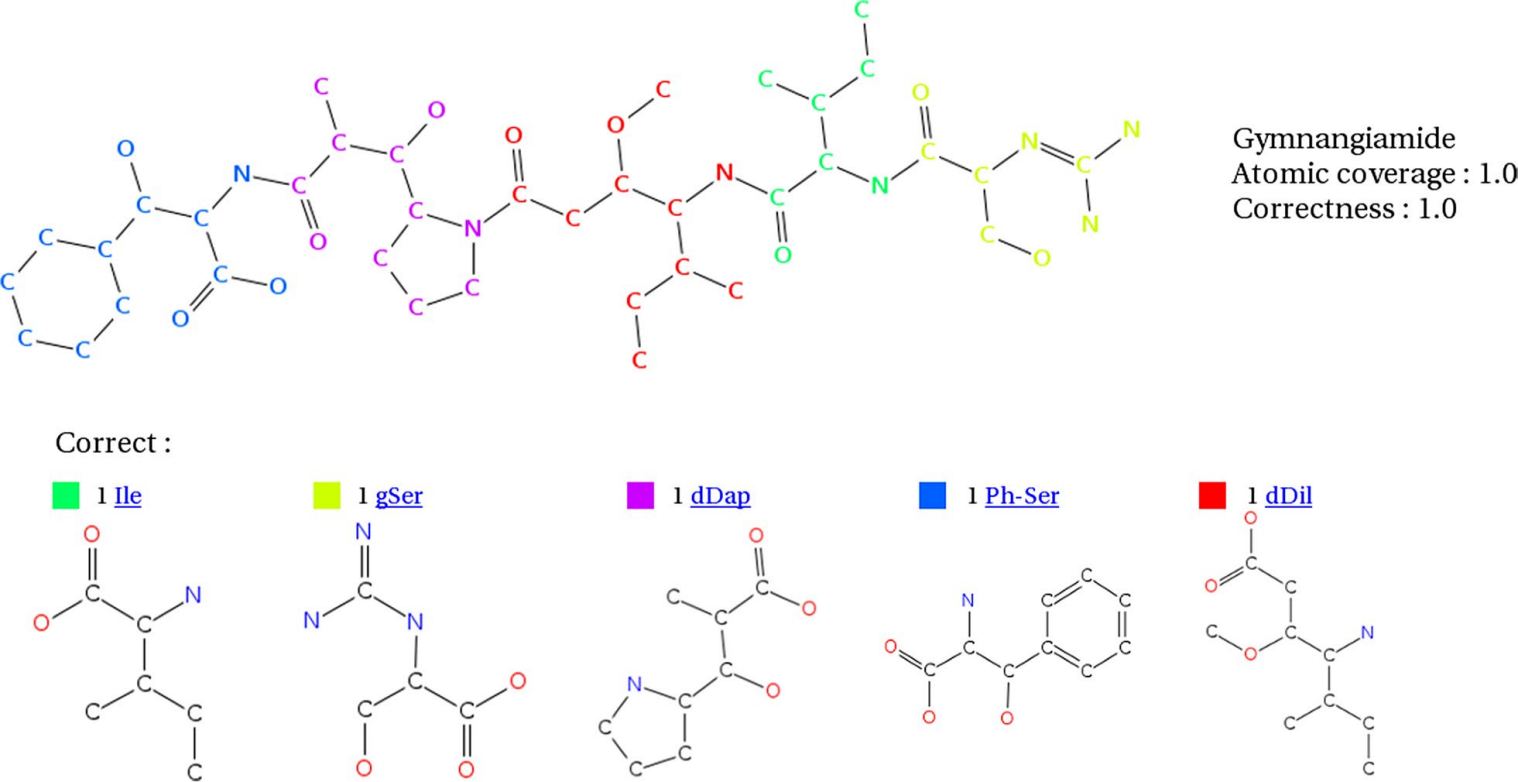
Gymnangiamide analysed by s2m after SMILES corrections. With the accurate SMILES for gSer, s2m was able to cover completely the polymer with the accurate monomers

It is important to note that the FP and FN classes are caused not only by wrong predictions of s2m, but also by errors in the tested datasets. Indeed, s2m allowed us to detect wrong annotations of monomeric structures or wrong SMILES for polymers or monomers. Figure 5 shows an example of a wrong annotation on Norine corrected by the analysis of the s2m results. Four of the five monomers are correctly mapped on the pentapetide. They represent 83 % of atoms correctly assigned over all the atoms of the polymer. The coverage rate is even higher: 96.2 % of the atoms are within a mapped monomer. Because of a wrong SMILES in Norine, the gSer monomer is not recognized and the tool tries to fill in the uncovered region with smaller candidate monomers like NMe-Ser. With the correct SMILES for gSer, s2m correctly recognizes all the monomers (see Fig. 6). We are currently overviewing the obtained results to update the annotations stored in the Norine database. So far, the rates for TP classes have been underestimated and should increase while the remediation process progress.

**Fig. 7:**
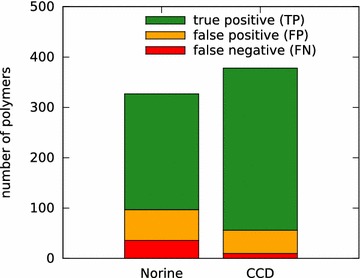
s2m succeed to predict correctly more than 3 / 4 of the tested polymers. Among the 327 polymers of Norine, 230 are correctly predicted (TP), 61 are totally covered but with few wrong monomers (FP) and 36 are not totally covered (FN). And among the 378 tested polymers of CCD, 322 were correctly predicted (TP), 46 aretotally covered but with few wrong monomers (FP) and 10 are not totally covered (FN)

At the time of writing of the article, 291 of the 327 peptides (89 %) of Norine and 368 of the 378 compounds (97 %) of CCD are totally covered. Among the partially covered polymers, only 1 compound of CCD has a coverage of 0.77, 2 peptides of Norine have a coverage of 0.8, while 34 peptides and 9 compounds have a coverage of 0.9. In total, the coverage reaches an average of 0.996 for Norine and 0.998 for CCD. So s2m succeeds in calculating monomeric structures that cover totally or at least 3 / 4 of the atomic structure of the tested polymers. Moreover, 230 peptides of the 327 (70 %) of Norine and 322 compounds of the 378 (85 %) of CCD are fully recognized (correctness of 1) by s2m. The correctness reaches an average of 0.934 for Norine and 0.953 for CCD. The counts are illustrated in Fig. 7. This represents a recall (or sensitivity) of 0.865 for Norine and 0.970 for CCD (0.919 for both sets) and a precision of 0.790 for Norine, 0.875 for CCD (0.830 for both sets). As previously mentioned, these rates are underestimated as the FP and FN rates are partially caused by errors in the databases.

### s2m execution time depends on technical issues

Notice that we distinguish the algorithm execution time and the complete execution time. The first one focuses on the monomer search and tiling parts, our own contributions. The second one includes the loading and post processing (the images generation for example) parts, which use CDK library.

First we discuss the algorithm execution time. The indexation of the monomer databases with our isomorphism algorithm coupled to an efficient and accurate tiling strategy ends with a fast tool. Indeed, the average algorithm execution time of s2m is 21 ms per polymer for Norine and only 7 ms per polymer for CCD. The difference in the execution time can be explained by the difference in topology and in the average size of the polymers (76 non-hydrogen atoms in Norine and 36 in CCD). The longest execution times are caused by polymers that need the run of the light search for tiling optimization.

On the web server dedicated to NRPs, the complete execution time is longer. We estimated the time with the example provided in the web server. The results are obtained after 3–5 s depending of the network latency. The huge difference between the algorithm execution time and the complete execution time on the web server can be explained by a few technical and program issues. First, the drawing of huge atomic structures is time consuming due to the difficulty of positioning harmoniously the atoms and their bonds. Second, the complete reloading of the jvm and the complete loading of our data for each query increase the latency period. We are working on a solution using a memory cache for our data with a permanently loaded jvm to improve the performances. We hope to reduce the online delay to 1 s with all these technical features for the queries without image generation.

The time measurements were computed on a desktop computer with an intel core i5-3470 CPU at 3.20 GHz with 8 Gb of RAM dedicated to the Java Virtual Machine.

## Conclusion and perspectives

As demonstrated, s2m automatically creates *de novo* monomeric structures for polymers. Our tool, which implements a smart algorithm, is efficient in terms of sensitivity. On average and per polymer for the two tested databases, the results are outputted in 2 s with an average coverage rate of 0.997, and with an average correctness rate of 0.943 ($82~\%$ of the polymers are correctly annotated). The source code is available under GNU AFFERO licence (https://github.com/yoann-dufresne/Smiles2Monomers) and through a web server for the NRPs structure predictions.

With the help of the tool we discovered wrong annotations or wrong chemical structures in the tested databases. So, we started the remediation of Norine data, with the help of the automatically generated monomeric structures and by going back to the literature. This proves that s2m is an efficient and accurate tool for automatic verification of already annotated polymers or for helping the annotation of newly discovered polymers. For example, s2m is included in MyNorine, the tool to submit new peptides in Norine (http://bioinfo.lifl.fr/norine/my/). It could be included in the remediation process of other databases such as CCD and BIRD [3].

s2m already outputs adequate results, based on a given monomer database. For the moment, the uncovered regions are partially covered by smaller monomers of the database. With the light matching, modifications as additional functional groups can already be detected (lose of an hydrogen replaced by a functional group). But, s2m depends on the completeness of the monomer database relating to the type of the target polymer. We are currently thinking of implementing a functionality that proposes unknown monomers by using local MCS against known monomers in the uncovered regions. As we generate residues from monomers by applying chemical reaction rules, s2m can be extended to label the type of bonds between identified monomers. Finally, if the monomers and the bonds are clustered by polymer types (for example peptide or carbohydrate), once the monomeric structure is predicted, s2m can infer the polymer type. So, s2m can screen chemical databases (storing atomic structures) to generate the monomeric notation and assign the predicted polymer to its more probable type. This high-throughput technique will increase the filling-in of specialized databases such as Norine with external data and will help to add new and more reliable annotations to the original databases.

## Additional file


10.1186/s13321-015-0111-5Isomorphism algorithm details. This supplementary material describes in detail our method to search for a residue into a polymer.
