# Supplementary material for: Smiles2Monomers: a link between chemical and biological structures for polymers
Source: J Cheminform. 2015 Dec 29;7:62. doi: 10.1186/s13321-015-0111-5 (PMC4693424; doi:10.1186/s13321-015-0111-5)
Supplement: Supplementary file 1 — 10.1186/s13321-015-0111-5Isomorphism algorithm details. This supplementary material describes in detail our method to search for a residue into a polymer. [file 13321_2015_111_MOESM1_ESM.pdf]

Isomorphism algorithm details

Y.Dufresne, L.Noe, V.Leclère, M.Pupin

The aim of this supplementary material is to explain in detail our method to search for a residue into a polymer. We will distinguish the *pattern* of a molecule, and the *chains* that can be used to search for this pattern. The pattern is the whole residue (more generally the query graph) or any connected sub-graphs of atoms composing this residue. A chain is a succession of growing sub-patterns that generates a given pattern starting from a single atom (more generally a node). So, a given pattern of length k named p_k can be generated by several chains c. In our algorithm, we construct chains by adding connected atoms one by one, following the chemical bonds between successive atoms (more generally the edges between nodes).

In the example of Figure 1, the proposed pattern is $a{-}b{-}d$ and the possible chains that can be used to search for the pattern are the following:

$$\begin{array}{ccccccc} c & = & p_1 & \rightarrow & p_2 & \rightarrow & p_3 \\ c_\alpha & = & a & \rightarrow & a{-}b & \rightarrow & a{-}b{-}d \\ c_\beta & = & b & \rightarrow & a{-}b & \rightarrow & a{-}b{-}d \\ c_\gamma & = & b & \rightarrow & b{-}d & \rightarrow & a{-}b{-}d \\ c_\delta & = & d & \rightarrow & b{-}d & \rightarrow & a{-}b{-}d \end{array}$$

Note that only four chains are generated, as they are based on successive connected sub-graphs that link neighbor nodes. The sub-pattern $a{-}d$ is never observed because a and d are not linked together.

Our goal is to determine from the set of chains of a given pattern, the one that minimizes the search time. The search time first depends on the frequency of the sub-patterns in the target polymer type and on the number of edges (more generally the arity) that should be explored to find a given pattern. Indeed, the more a sub-pattern is frequent, the more time is spent to find all its occurrences. Once a sub-pattern is found, the remaining edges starting from a specific node of this sub-pattern should be explored to continue the construction of the pattern, according to a given growing chain.

For example in Figure 1, the node a has an arity of 3. So, the 3 edges starting from a should be explored to find the next node prompted by the growing chain. In the case of the chain c_α, the sub-pattern $a{-}b$ is constructed. Then, two remaining edges must be explored from b to find d and construct the given pattern $a{-}b{-}d$

Greedy algorithm

The greedy algorithm chooses the most selective node at each step of the chain construction. First, the frequency of each node label is calculated by counting every types of labels in the learning database. The most selective label, i.e. the one with the lower number of occurrences, is the starting point for chains of patterns containing this label. Then, its most selective neighbor is recursively added to the growing chain. The goal is to constantly minimize the number of initial solutions.

For example in Figure 1, the most selective node label is a with a 2/18 frequency, so the greedy algorithm will start to search for a and then recursively determine its most selective neighbor. In this case, only one chain among the four chains generated from the studied pattern starts with a, this is $c_\alpha = a \rightarrow a{-}b \rightarrow a{-}b{-}d$. So, the greedy algorithm outputs the chain c_α as the most selective one.

But this chain selection is greedy: the selectivity is calculated for each node independently of its links to other nodes. It does not guaranty to have the most selective chain that takes into account the previous steps of the chain construction.

Markovian chains

Markovian chains select the best succession of sub-patterns that constructs the most selective chain. In other words, they allow to construct the chain that minimizes the global search time. First, the time needed to find each possible chain constructed from a given pattern is estimated. Then, the chain requiring the minimal time is selected.

For a searched pattern p_k of size k and a given chain c, the full estimated time T spent to search for a succession of growing patterns $p_1, p_2, \ldots$ until p_k is given by:

$$T(c) = T(p_0 \rightarrow p_1 \rightarrow \ldots \rightarrow p_k) = \sum_{i=1}^{k} T(p_{i-1} \rightarrow p_i)$$

where $T(p_{i-1} \rightarrow p_i)$ is the time spent extending the p_{i-1} pattern to the p_i pattern. It is to notice that p_0 is here the empty pattern ϵ which is not searched. The initial time $T(p_0 \rightarrow p_1)$ spent to search for p_1 from the learning database of polymers is proportional to N, where N is the full number of nodes in the learning database.

By recursion, once the search for p_{i-1} gives $n_{p_{i-1}}$ occurrences (which can, in some cases, be exponentially $>> N$), the next search for p_i will give n_{p_i} occurrences, and is of time :

$$\forall i \geq 2 \qquad T(p_{i-1} \rightarrow p_i) = n_{p_{i-1}} \times a_{p_{i-1} \rightarrow p_i} \times t$$

where $a_{p_{i-1} \rightarrow p_i}$ is the remaining arity of the node from which we wish to extend p_{i-1} into p_i, and t the time to compute a comparison between two labels. The time t can be maximized by a constant, so it can be withdrawn from the formulas

We introduce a filtering ability f_i defined by

$$f_{p_{i-1}\to p_i} = \frac{n_{p_i}}{n_{p_{i-1}}}$$

and when cumulated

$$n_{p_i} = N \times \prod_{j=1}^{i} f_{p_{j-1}\to p_j}$$

Notice that $f_{p_{j-1}\to p_j}$ is naturally bounded by the arity of the node extended during the construction of p_j from p_{j-1}. Now we can introduce f in $T(c)$ calculation.

$$\begin{aligned}
T(c) &= T(p_0 \to p_1) + \sum_{i=2}^{k} \big(a_{p_{i-1}\to p_i} \times N \times \prod_{j=1}^{i-1} f_{p_{j-1}\to p_j}\big) \\
T(c) &= N + N \sum_{i=2}^{k} \big(a_{p_{i-1}\to p_i} \times \prod_{j=1}^{i-1} f_{p_{j-1}\to p_j}\big) \\
T(c) &\propto_N 1 + \sum_{i=2}^{k} \big(a_{p_{i-1}\to p_i} \times \prod_{j=1}^{i-1} f_{p_{j-1}\to p_j}\big)
\end{aligned}$$

For example in Figure 1, the estimated computational time $T(c_\alpha)$ on the previously mentioned chain c_α is the following:

$$\begin{array}{rcccccccc}
c_\alpha &=& \epsilon \to & a & \to & a{-}b & \to a{-}b{-}d & & \\
T(c_\alpha) &=& T(\epsilon \to a) &+& T(a \to a{-}b) &+& T(a{-}b \to a{-}b{-}d) & & \\
&\propto_N& 1 &+& 3 \times \frac{2}{18} &+& (3-1) \times \frac{2}{18} \times \frac{3}{2} & & \\
&=& 1 &+& \frac{1}{3} &+& \frac{1}{3} &=& \frac{5}{3}
\end{array}$$

Note that c_α is a possible chain to search for $a{-}b{-}d$, however not the best one. In practice, we select the most selective chain for each pattern, applying the following formula:

$$T(p_k) = min_{\forall c \,|\, c = p_0 \rightsquigarrow p_k}\big(T(c)\big)$$

For example in Figure 1 , the most selective chain for the pattern $a-b-d$ is c_δ (with $T(c_\delta) = \frac{14}{9}$) because the extension of the sub-pattern $b-d$ to the pattern $a{-}b{-}d$ has an estimated search time smaller than the extension of the sub-pattern a–b, and the extension of the sub-pattern d has an estimated search time smaller than the extension of the sub-pattern b.

Since the set of chains $c = p_0 \rightsquigarrow p_k$ used to search a pattern p_k (or in more general case, any set of patterns) can be represented by a Directed Acyclic Graph (DAG), the dynamic programming aspect of the computation of $T(p_k) = min_{\forall c \,|\, c = p_0 \rightsquigarrow p_k}\big(T(c)\big)$ can be easily established. It has two consequences: (a) only one optimal chain per pattern in the DAG must be kept, and the full set of optimal chains for all patterns can itself be represented by a tree in a very compact way ; (b) the p_k pattern(s) and the optimal chain to find it(them) efficiently can be set by a classical memory efficient approach computing the p_i patterns from the p_{i-1} already established optimal chains (for all $i \in [1..k]$), with emphasis on removal of all non optimal chains.

Hybrid algorithm

We define two possible algorithms to choose the best chain of any sub-pattern to minimize its search time. On the one hand, the greedy algorithm computes rapidly (polynomial time) the best chains for each residue of the database but can fall in local minima. On the other hand, the Markovian model computes, at a slower rate (exponential time) the best chains because it finds global minima, which means a guaranty of optimality for several critical residues. To take advantage of both models, we implemented an hybrid solution using the Markovian model on the m first extensions of the chain and finishing next extensions with the greedy model. The Markovian model applied only on the first m steps avoids critical exponential numbers of intermediate solutions.

Results

We tested the performances of our algorithm on the Norine dataset. We measured only the search time for all the monomers on each polymer of the database. We compared the search time for a random order, for the greedy algorithm (Markovian with $k = 1$) and for several values of k in our Hybrid Algorithm. We obtained an average execution time per polymer of 7.8 ms for aed random order, 7 ms for the greedy algorithm and we reach a plateau for $k = 3$ at 6.1 ms. So, the greedy algorithm reduces time by 10% in comparison to a random order. Moreover, when $k = 3$, the hybrid algorithm reduces time by 20%, also in comparison to a random order. The isomorphism algorithm used for SMARTS matching in CDK uses an ordering only for the very first atom search, which is better than the random algorithm but worse than the greedy one. In comparison with the CDK SMARTS algorithm, we improved the isomorphism time from 10% up to 20%. The larger the number of monomers in the database is, the more efficient the search is, compared to the loading and preprocessing overheads (IO + SMILES parsing).

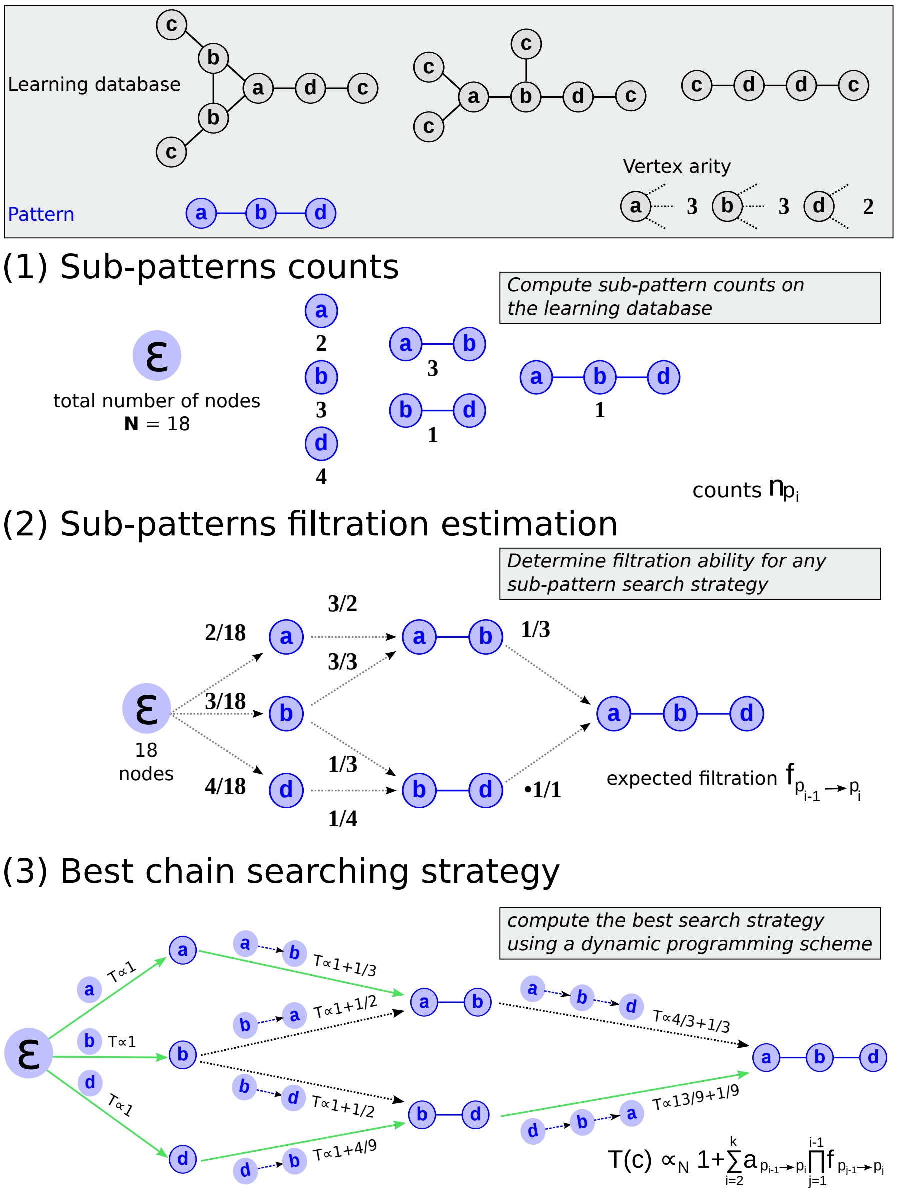

Figure 1: **Index creation using the Markovian method.** This figure represents the full process to build a Markovian index. The gray rectangle contain an example of learning database and, in blue, the pattern that we want to index. (1) Counts of the sub-patterns in the learning database: computation of the number of occurrences of each sub-pattern of $a - b - d$ in the learning database. (2) Estimation of the sub-patterns filtration: computation of the number of occurrences of a sub-pattern over the counts for the previous sub-pattern. (3) Selection of the best chain for the pattern: computation of the expected computing time for each sub-pattern and selection of the faster chain at each step. The selected paths in the Directed Acyclic Graph is highlighted in green.
